# Supplementary figures and images for: Evaluation of data driven low-rank matrix factorization for accelerated solutions of the Vlasov equation
Source: PLoS One. 2025 Jun 9;20(6):e0325304. doi: 10.1371/journal.pone.0325304 (PMC12148180; doi:10.1371/journal.pone.0325304)

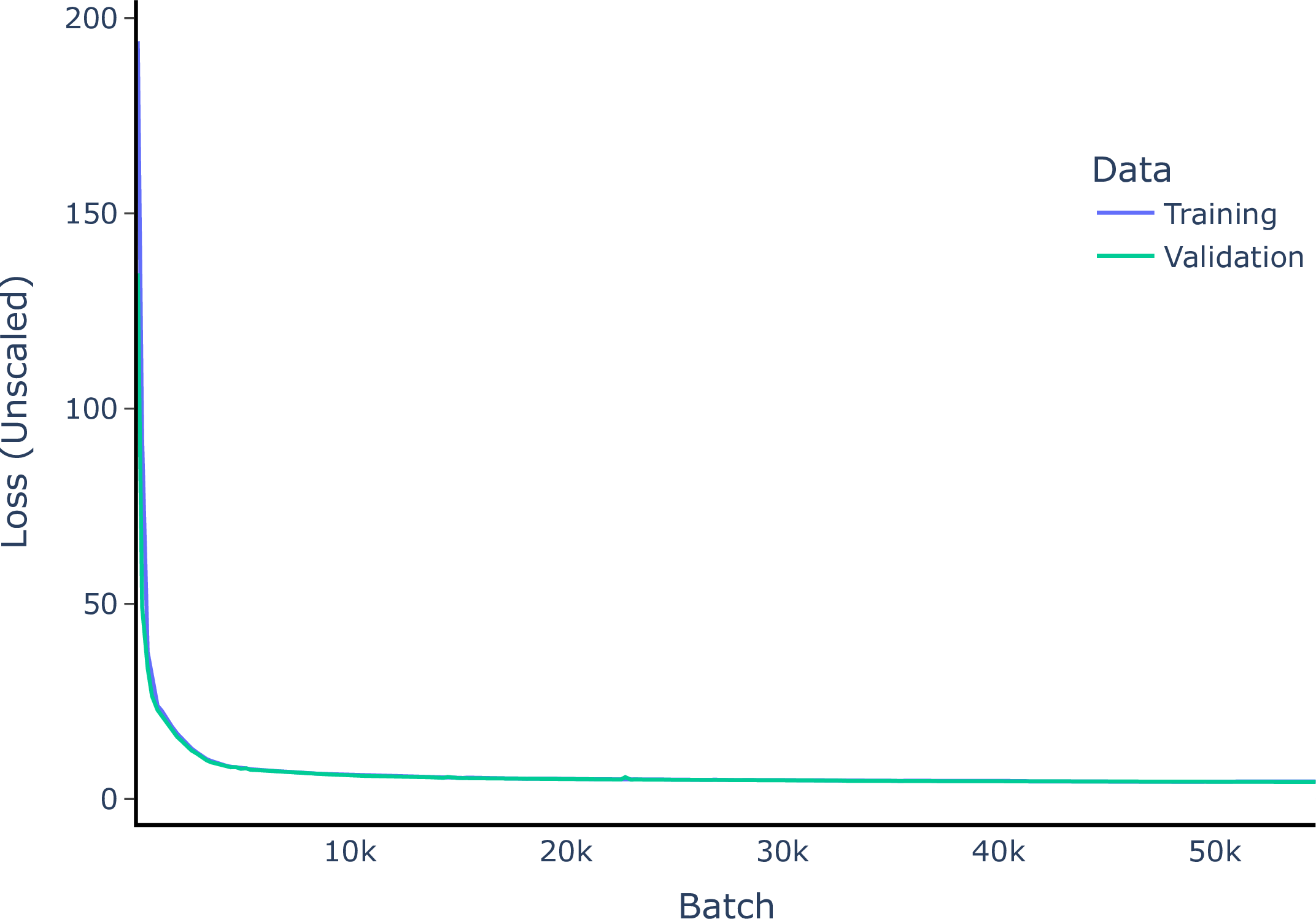

Supplement: S1 Fig — The training and validation loss curve for the best network tested (ConvMF), with loss being unscaled by norm (TIFF) [file pone.0325304.s001.tif]
